# Anodal tDCS Enhances Verbal Episodic Memory in Initially Low Performers

**DOI:** 10.3389/fnhum.2017.00542

**Published:** 2017-11-07

**Authors:** Annegret Habich, Stefan Klöppel, Ahmed Abdulkadir, Elisa Scheller, Christoph Nissen, Jessica Peter

**Affiliations:** ^1^University Hospital of Old Age Psychiatry and Psychotherapy, University of Bern, Bern, Switzerland; ^2^Faculty of Biology, University of Freiburg, Freiburg, Germany; ^3^Department of Psychiatry and Psychotherapy, Faculty of Medicine, University of Freiburg, Freiburg, Germany; ^4^Department of Computer Science, University of Freiburg, Freiburg, Germany; ^5^University Hospital of Psychiatry and Psychotherapy, University of Bern, Bern, Switzerland; ^6^Department of Neurology, Inselspital, University of Bern, Bern, Switzerland

**Keywords:** transcranial direct current stimulation, verbal episodic memory, word list learning, dorsolateral prefrontal cortex, response variability

## Abstract

The left dorsolateral prefrontal cortex (DLPFC) is involved in encoding and retrieval of episodic memories, and thus, is frequently targeted in non-invasive brain stimulation paradigms, aiming for its functional modulation. Anodal transcranial direct current stimulation (tDCS), that boosts neuronal excitability in stimulated cortical areas, has been found to increase cognitive skills differentially, depending on the initial performance. We hypothesize that the benefit of tDCS on verbal episodic memory can be extrapolated from the participants’ baseline performance. In the present randomized, double-blind, parallel group study, healthy young adults (*n* = 43) received either real anodal or sham tDCS over their left DLPFC during the encoding phase of a verbal episodic memory task. Forty words were presented visually thrice with immediate vocal retrieval after each block and an additional delayed recall. We conducted a moderation analysis to test the modulating effect of initial episodic memory retrieval, adjusted for primacy and recency effects, on delayed recall under real or sham stimulation. Despite the absence of a significantly beneficial tDCS effect at the group level, we found that the number of remembered midlist words in the first retrieval significantly moderated the stimulation effect in such a way that initially low performers experienced the highest gain from real stimulation. These results suggest that anodal tDCS to the left DLPFC improves memory functions only so far. While only marginal stimulation-induced gains occur in cognitively unimpaired populations, greater stimulation benefits might be expected in individuals with clinically relevant deficiencies in the verbal episodic memory domain.

## Introduction

Episodic memory allows the encoding, storage, and retrieval of information connected to specific events on a personal timeline (Tulving, 2002). While the hippocampus is strongly involved in these processes (Schott et al., 2013; Sneve et al., 2015), functional neuroimaging studies (Fletcher, 2001; Spaniol et al., 2009; Manenti et al., 2010) and transcranial magnetic stimulation research (Miniussi et al., 2003; Gagnon et al., 2010, 2011) have revealed that the activation of the dorsolateral prefrontal cortex (DLPFC) is another prominent feature during the encoding and retrieval of verbal episodic memory contents. The respective processes involve the two hemispheres of the DLPFC in an asymmetric manner, the left DLPFC being crucial for encoding whereas the right DLPFC is predominantly associated with retrieval (Shallice et al., 1994; Sandrini et al., 2003). Consequently, the DLPFC is commonly targeted in non-invasive brain stimulation (NIBS) studies, aiming to boost cognitive functions, especially episodic memory.

One of those NIBS techniques, namely transcranial direct current stimulation (tDCS), relies on the passing of electrical current into the brain, resulting in polarity-dependent tonic changes (i.e., depolarisation and hyperpolarization) of the resting membrane potential of neurons in the targeted brain area (Nitsche et al., 2003). Notwithstanding that anodal stimulation increases excitability whereas cathodal stimulation decreases excitability in the targeted brain area, behavioral outcomes of tDCS over the DLPFC during the encoding of verbal episodic memory tasks have been highly variable. Applied over the left DLPFC, anodal tDCS improved the rate of word list learning in healthy young participants (Nikolin et al., 2015) as well as increased the performance of elderly individuals during the delayed recall of the learned items (Sandrini et al., 2016). However, another study challenged the general enhancing effects of anodal tDCS, as its application resulted in a decreased recognition of verbal and non-verbal material (Manuel and Schnider, 2016).

Apart from contradictions between separate studies, which could be attributed to the high variability in experimental designs (e.g., stimulation duration and timing, current density, stimulation site), there is also a large heterogeneity in individuals’ responsiveness within single studies (Krause and Cohen Kadosh, 2014). In addition to physiological states [e.g., motor-evoked potential latencies (Wiethoff et al., 2014)], baseline skills in different modalities [e.g., fine motor control (Furuya et al., 2014), spatial visual acuity (Reinhart et al., 2016), reading efficiency (Turkeltaub et al., 2012)], were found to bias the stimulation gain in the investigated task, to the extent that low performers were more likely to profit from the stimulation compared to high performers who were even negatively affected (Rosen et al., 2016). Until now, this aspect of conditional tDCS effects has been left unacknowledged in studies concerned with the stimulation-induced improvement of episodic memory.

Therefore, the present study investigated whether initially low performing individuals are more likely to benefit when receiving anodal tDCS over their left DLPFC during the encoding phase of a verbal episodic memory task as compared to initially high performers, in accordance with previous observations in other modalities. More precisely, we tested to what extent an individual’s initial verbal episodic memory performance, adjusted for primacy and recency effects, moderated the tDCS effect on a subsequent delayed recall. This analysis might provide valuable insights into the question of, who profits from the stimulation, thus enabling the identification of suitable subjects prior to the intervention.

## Materials and Methods

### Participants

Forty-four healthy young adults were recruited for the study. Due to technical problems data were lost for one participant, leaving the data sets of forty-three healthy, right-handed [gauged via the Edinburgh Handedness Inventory, EDI; *LQ* > 50; (Oldfield, 1971)] participants [aged 24.8 ± 2.9 years (mean ±*SD*), range: 20–30 years; 22 females; at least 12 years of education] for further evaluation. All participants gave written informed consent prior to the experiment and were reimbursed with 24 euros for their participation. The study was approved by the ethics committee of the University of Freiburg (reference number: 561/15) and complies with the Helsinki Declaration.

### Inclusion and Exclusion Criteria

Prior to enrolment, all participants were screened on the telephone and only invited to the study if deemed eligible. All participants were native German speakers, non-smokers, with normal or corrected-to-normal vision and no history of psychiatric or neurological disorders. Further exclusion criteria were past head injuries, metal implants in the head-area, seizures, current or life-time alcohol or drug abuse, intake of psychotropic drugs, pregnancy and skin diseases like neurodermatitis. Additionally, participants with relevant depressive symptoms [according to the Beck Depression Inventory II (BDI II) > 13; (Beck, 1961)], as assessed the day before the study via a previously emailed questionnaire, were excluded from the study. To ensure comparable verbal intelligence scores, participants completed the German vocabulary test WST (Herzfeld, 1994) during the on-site visit.

### Study Procedure

**FIGURE 1:**
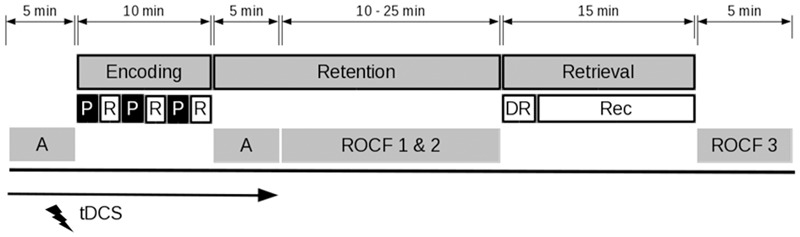
Study procedure. Following an alertness task (A) participants performed a verbal episodic memory task. Encoding consisted of the presentation (P) and immediate recall (R) of forty words in three successive rounds. During the retention interval, the attention task was repeated and participants copied and immediately retrieved the Rey–Osterrieth complex figure (ROCF). In the retrieval phase, participants performed a delayed recall (DR) and a recognition task (Rec). Subsequently, there was a delayed recall of the Rey–Osterrieth complex figure. The application of tDCS was restricted to the initial attention task and the encoding phase.

In this double-blind, sham-controlled, parallel group study all participants were tested individually in a single session that lasted for approximately 1.5 h. They were randomly assigned to one of two groups (real or sham tDCS). The session was divided into three phases, namely encoding, retention, and retrieval of a verbal episodic memory task (**Figure 1**). Participants received anodal tDCS over their left DLPFC during encoding. During the retention interval participants copied and drew the Rey–Osterrieth complex figure (Shin et al., 2006) to prevent active rehearsal of the encoded words. Since the performance in this task primarily relied on visuospatial functions, it was deemed sufficiently different to not interfere with the delayed recall of the previously learned word list.

### Stimuli

Eighty nouns were selected from parallel versions of the revised California Verbal Learning Test (CVLT-II; Delis et al., 2000) and a set of potentially emotionally connoted words (Herold, 2008). The words were chosen with regard to the phonemic, semantic, and emotive requirements of a coordinated tDCS study on implicit learning. We chose two to three times more words than previous studies (Elmer et al., 2009; Nikolin et al., 2015) to lower the risk of ceiling effects that could have precluded the emergence of more sizable stimulation benefits. Forty words were used for encoding and an additional forty words were randomly intermingled during the recognition task. In a pilot study with ten participants, we affirmed that the chosen number of words was sufficient in order to avoid ceiling effects.

### Experimental Schedule

The experiment was computerized and programmed in Presentation^®^ software (Version 18.1, Neurobehavioral Systems, Inc., Berkeley, CA, United States). The participants were seated in front of a 14-inch computer screen at a distance of approximately 0.5 m and performed the experiment in a well-lit, quiet room. Following the mounting of the electrodes (further information is provided in section tDCS), the stimulation was started simultaneously with the first block of an attention task (5 min) in which the participants were required to respond to the appearance of a single white cross on a black screen, either preceded by an auditory cue or not (i.e., phasic and intrinsic alertness: **Figure 1**). During this time span, the participants could accustom themselves to the tingling sensation associated with the ramp up phase of tDCS. Moreover, previous research suggested that anodal tDCS effects on cortical excitability arise after 5 min of stimulation (Nitsche and Paulus, 2000). After the attention task, participants were informed that they would need to memorize a set of words to the best of their ability. During the encoding phase, the words were presented on the computer screen in white against a black background in a randomized order in three successive blocks, each word appearing once in each block. After a priming fixation cross, presented for 200 ms, each word remained on display for 1 s and was followed by a blank screen of a randomized duration between 0.5 and 2.5 s. After each block, participants were asked to orally retrieve as many words as possible within 2 min. The answers were recorded in separate audio files. During the retention interval of approximately 20 min, participants completed the prior attention task a second time as well as copied and immediately recalled the Rey–Osterrieth complex figure (Shin et al., 2006; **Figure 1**) in a self-paced manner. In the retrieval phase, participants first performed a free delayed recall of the memorized words and then completed a recognition task. Therein, participants were asked to indicate, by pressing a button, whether each of the eighty presented nouns belonged to the list of initially memorized words or was deemed a new word (i.e., distractor). After having completed the recognition task, participants were asked to draw the Rey–Osterrieth complex figure once again from memory (delayed recall). Performance in the episodic memory task was measured in terms of an absolute number of correctly recalled words during free retrievals as well as by means of reaction times and the proportion of correct responses in the recognition part. Alertness was evaluated according to reaction times.

In conformity with the questionnaire proposed by Brunoni et al. (2011), we enquired about perceived side effects of the stimulation and controlled the consistent blinding of the participants with respect to the stimulus condition.

### tDCS

Transcranial direct current stimulation was delivered by a battery-driven DC-Stimulator PLUS (NeuroConn GmbH, Ilmenau, Germany) using a pair of saline-soaked sponge electrodes (5 cm × 7 cm). The anode was placed over the left DLPFC, centered over the F3 position corresponding to the 10–20-EEG system of electrode placement (Klem et al., 1999). The contralateral supraorbital area (above the right eyebrow) was selected as the reference electrode position. A person not involved in the data collection (JP) allotted the codes for sham or real anodal tDCS, thus providing an effective blinding for both participant and experimenter. Real anodal tDCS consisted of a 15 s ramp up phase after which the current remained constant at 1 mA for 20 min and was ramped down for another 15 s afterward. Total current density did not exceed 0.03 mA/cm^2^ at any point in time and thus remained below safety limits (Poreisz et al., 2007). During the sham stimulation, the current was ramped down after 30 s to ensure the best possible blinding of the participant with regard to the stimulus condition. Thus, participants experienced the same itching sensation associated with the onset of real anodal tDCS without eliciting stimulation effects that outlasted 30 s (Gandiga et al., 2006).

### Statistical Analysis

All data analyses were carried out in SPSS (Version 23.0, IBM Corp., Armonk, NY, United States) using parametric (whenever Kolmogorov–Smirnov tests indicated no violation of the normality assumption) or non-parametric tests and *p <* 0.05 denoted statistical significance. Aside from the moderation analysis, all tests are purely exploratory to rule out the possibility of other group differences driving the observed effects.

#### Verbal Episodic Memory Task

**FIGURE 2:**
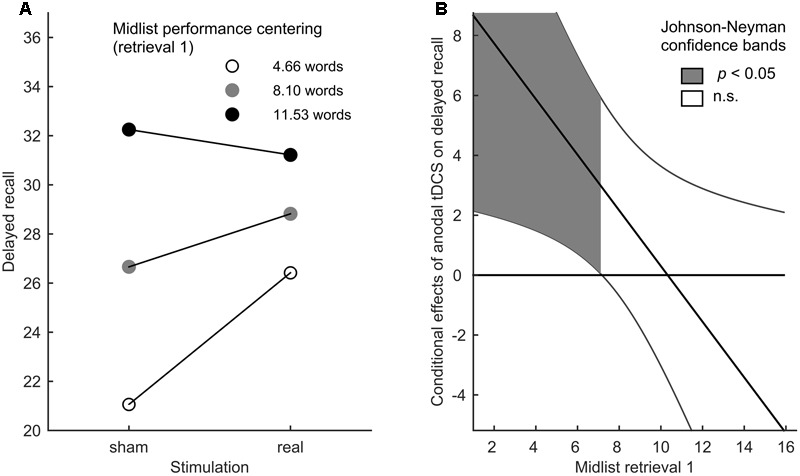
Differential tDCS benefits. **(A)** Moderation effect of midlist performance in retrieval 1 on the relation between stimulation and delayed recall performance for three performance centerings in retrieval 1 (–SD, mean, +SD). **(B)** Conditional effects of performance in the delayed recall. Johnson–Neyman confidence bands indicate the midlist performance range in retrieval 1 in which the latter has a significant predictive value.

As we were primarily interested in moderating effects of baseline episodic memory performance on tDCS gains in the delayed recall, we applied the SPSS PROCESS macro (Version 2.16) (Hayes, 2013) to perform a moderation analysis with stimulation (real or sham) as the focal predictor, midlist performance in retrieval 1 as the moderator variable and delayed recall performance as the outcome variable. Therein we investigated whether the initial performance in the first retrieval affected the benefit of tDCS on the delayed recall. On the whole, a moderation analysis resembles a multiple regression analysis with interaction terms in as much as the effect of the predictor on the outcome variable is conditional, i.e., it differs depending on the value of the moderator (Jaccard and Turrisi, 2003). To facilitate the interpretation of interaction effects, the impact of the continuous moderator on the predictive value of tDCS is evaluated at three centerings (-SD, mean, +SD), corresponding to low, moderate, and high baseline performance (**Figure 2A**). By using the Johnson–Neyman technique (Bauer and Curran, 2005; Hayes, 2013), the interaction effects can be further probed to reveal the performance range in which stimulation has a significantly positive or negative predictive value (**Figure 2B**).

Considering that different list segments represent distinct cognitive abilities, we chose recalled midlist items as a reliable measure of episodic memory performance in the first retrieval (Krueger and Salthouse, 2011). Based on the finding by Murdock and Bennet (1962) that the number of words impacted by the serial position effect is independent of the length of the word list (i.e., the primacy effect extending over first 3 to 4 positions and the recency effect extending over last 8 items), positions 5 through 32 were considered to be midlist locations in the list of presented words. To assess overall group difference induced by the stimulation on the encoding trials a 2 × 3 repeated measures ANOVA was performed on the number of remembered words in each of the immediate retrievals (i.e., retrieval 1–3) with stimulation group as the between-subjects factor. Additionally, a two-tailed *t*-test was applied to test for tDCS-related differences in the number of remembered words in the delayed recall.

Data from the recognition task were tested for group differences in terms of the sensitivity index *d’* and criterion *c* as well as reaction times by means of Mann–Whitney *U* tests.

#### Rey–Osterrieth Complex Figure

We performed a 2 × 2 repeated measures ANOVA on the drawing scores achieved during immediate and delayed recall of the Rey–Osterrieth complex figure with stimulation group as the between-subjects factor.

#### Alertness Task

As previous studies (Clark et al., 2012; McKinley et al., 2012) suggested that tDCS effects on learning paradigms may be primarily driven by enhanced attention and vigilance induced by the stimulation, we also tested alertness for a stimulation impact. A 2 × 2 repeated measures ANOVA was calculated for reaction times pre- and post-stimulation with stimulation group as the between-subjects factor. Pearson correlations were conducted to test for a relationship between alertness and performance in any of the retrievals.

## Results

Statistical tests revealed no significant differences regarding age, years of education, BDI-II scores, handedness indices, or verbal IQ between the two stimulation groups (**Table 1**). The stimulation was generally well tolerated. Tingling (81.4%), burning sensation (44.2%), erythema (37.2%), and itching (34.9%) were the most commonly reported sensations, manifesting with mild to severe intensities. Sham and anodal stimulation did not significantly differ in any of the perceived side effects. Moreover, forced guessing as to the respective group assignment subsequent to the stimulation was at chance level for both participants [Pearson’s χ^2^(1) = 0.054, *p* = 0.817] and examiner [Pearson’s χ^2^(1) = 1.311, *p* = 0.525].

**Table 1:** Demographic characteristics of participants grouped according to stimulation (mean ± SD).

	Sham (*n* = 21)	Real (*n* = 22)	*p*-value
Gender	11 female	11 female	0.88 (χ^2^)
Age (years)	25.14 ± 3.26	24.55 ± 2.56	0.51 (*t*)
Education (years)	16.24 ± 2.47	15.93 ± 2.48	0.87 (*U*)
BDI II (0–63)	3.38 ± 3.35	4.64 ± 3.80	0.25 (*U*)
WST (0–42)	31.81 ± 2.82	32.68 ± 2.48	0.28 (*U*)

### Verbal Episodic Memory Task

**FIGURE 3:**
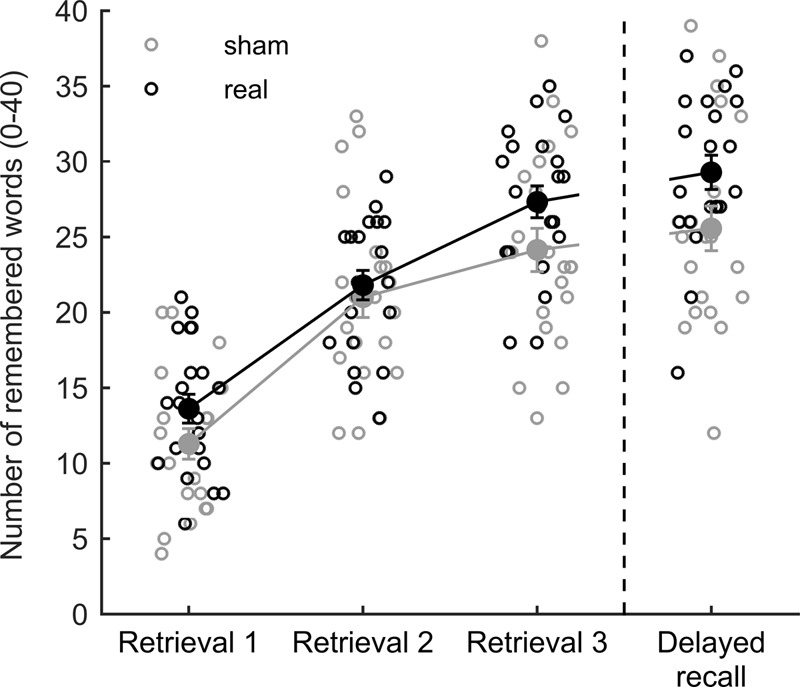
Performance in the verbal episodic memory task during encoding and retrieval phase. Each circle represents the performance of a single subject while large dots denote mean values with standard errors for each stimulation group.

The overall fit of our moderation model reached significance [*F*_(3,38)_ = 13.985, *p* < 0.001, *R*^2^ = 0.525, Cohen’s *f*^2^ = 1.105], indicating a predictive value of initial performance on stimulation impact in the delayed recall insofar as initial low performers experienced the highest gain from tDCS (**Figures 2A,B**). In this model, the simple effects of initial midlist performance [*t*_(38)_ = 5.358, *p* < 0.001] and stimulation [*t*_(38)_ = 2.662, *p* < 0.001] as well as their interaction [*t*_(38)_ = -2.232, *p* = 0.032] were found to be significant. The latter also led to a significant increase in explained variance [*R*^2^_change_ = 0.062, *F*_(1,38)_ = 4.980, *p* = 0.032]. By using Johnson–Neyman confidence bands (Johnson and Neyman, 1936; Bauer and Curran, 2005; Hayes, 2013) it became evident that the positive predictive value of the initial performance on the stimulation effect was restricted to those participants who recalled less than or equal to seven midlist words in the first retrieval (**Figures 2A,B**), which pertains to 44.2% of the tested sample. In the absence of a significant overall stimulation effect [*F*_(2,39)_ = 2.161, *p* = 0.149] on retrieval 1–3, the 2 × 3 repeated measures ANOVA merely revealed a significant effect of retrieval round [*F*_(2,39)_ = 222.659, *p* < 0.0005]. A *t*-test on the delayed recall performance showed no significant effect of tDCS [*t*_(40)_ = -1.991, *p* = 0.053, **Figure 3**].

Irrespective of the stimulation group, participants showed ceiling effects in the recognition task with regards to percentages of correct answers [93.7 ± 5.9% (mean ±*SD*)] while reaction times for correct responses showed a substantial distributional skewness [1770 ± 549 ms (mean ±*SD*)]. Further tests revealed no significant differences between either sensitivity index *d’* (*U* = 214.500, *p* = 0.688), criterion *c* (*U* = 201.500, *p* = 0.467) or reaction times (*U* = 178.500, *p* = 0.291) in the recognition task between the two stimulation groups.

### Rey–Osterrieth Complex Figure

Participants’ drawing scores did not significantly differ between the two retrieval rounds [*F*_(1,40)_ = 0.036, *p* = 0.851] nor did stimulation have any impact [*F*_(1,40)_ = 0.036, *p* = 0.850].

### Alertness Task

A 2 × 2 repeated measures ANOVA showed that even though reaction times generally improved between the two blocks of the alertness task in both phasic [*F*_(1,40)_ = 4.308, *p* = 0.044] and intrinsic alertness [*F*_(1,40)_ = 4.925, *p* = 0.032] there was no significant main effect for stimulation for phasic [*F*_(1,40)_ = 0.930, *p* = 0.341], or intrinsic alertness [*F*_(1,40)_ = 0.384, *p* = 0.539]. Likewise, no significant interaction emerged between time and stimulation for either phasic [*F*_(1,40)_ = 0.046, *p* = 0.831] or intrinsic alertness [*F*_(1,40)_ = 1.040, *p* = 0.314] (**Table 2**).

**Table 2:** Response times in alertness task (mean ± SD) prior to and after encoding phase of verbal episodic memory task.

	Intrinsic		Phasic	
	Real	Sham	Real	Sham
Pre	307 ± 31 ms	316 ± 25 ms	301 ± 24 ms	310 ± 42 ms
Post	304 ± 30 ms	306 ± 32 ms	294 ± 22 ms	302 ± 35 ms

Additionally, none of the calculated correlation analyses evidenced a significant correlation between alertness, both phasic and intrinsic, and performance in any of the retrievals (all *p* ≥ 0.1).

## Discussion

Our study demonstrates that, even in a homogenous sample of healthy young individuals, stimulation-induced benefits on verbal episodic memory recall are predicated upon individual baseline performance, with initial low performers profiting the most from anodal tDCS.

By increasing the number of presented words in the encoding phase to 40, we successfully avoided ceiling effects, which may have reduced the probability of detecting anodal stimulation effects during free recall in previous studies (Elmer et al., 2009; Nikolin et al., 2015). However, no significant overall gains due to tDCS substantiated during the delayed free recall of learned words, but episodic memory performance rather improved in a more differential manner as only initially low performers responded in the anticipated direction following real anodal tDCS. Reports of similar findings pervade the tDCS literature (Turkeltaub et al., 2012; Furuya et al., 2014; Reinhart et al., 2016), even to the extent of opposed effects in different subgroups of the study population (Rosen et al., 2016), but none of them had centered on episodic memory. Previous studies in sensory and cognitive modalities split participants into two distinct groups according to their level of expertise in the examined task, differentiating between amateurs (i.e., untrained low performers) and skilled experts, either by direct recruitment into the subgroups or retrospective allocation. Furuya et al. (2014; *n* = 26) showed that the beneficial effects of anodal tDCS in the form of improved motor control were largely restricted to musically untrained individuals whereas the same intervention impaired timed-sequence finger movements in highly trained pianists. Apart from the motor domain, this discrepancy also pervades cognitive skills insofar as in a study by Turkeltaub et al. (2012; *n* = 22), the tDCS-induced improvement in reading efficiency was specific to below average readers. These findings indicate ceiling effects in individuals with already distinct competences in a given domain. This also corresponds to the observation of greater stimulation outcomes in clinical populations as compared to healthy controls (Brunoni and Vanderhasselt, 2014). However, even when refraining from this dichotomous division, inter-individual response variabilities persist within homogenous samples of trained musicians (Rosen et al., 2016) or healthy young volunteers without unique characteristics (Mayseless and Shamay-Tsoory, 2015; Reinhart et al., 2016) (all *n* ≤ 20) following the seemingly inherent principle of greater stimulation gains in individuals with lower baseline skills.

In the present study, we only included healthy young participants with advanced education levels (Schrauwen et al., 2014), forgoing a preselection regarding individual episodic memory performance. This approach allows a better representation of generally encountered abilities, which are spread out on a continuous scale rather than following a binary classification. On the other hand, it presented us with the issue of choosing a dependable measure for baseline performance in order to investigate its moderating influence on tDCS-induced gains in the delayed recall. Generally speaking, the measure used to rate initial performance needs to be as congruent as possible with the queried cognitive function as, even though a selective transfer of stimulation gains was demonstrated across different working memory domains (Au et al., 2016), the tDCS-induced improvements are thought to be mostly restricted to the task executed concurrently to tDCS (Bikson and Rahman, 2013), an approach that also warrants a greater focality of the stimulation. Here, we chose not to precede the episodic memory task proper with an assessment of baseline memory ability, first because a prior exposure to word list learning can interfere with the measurement and second, because different tests of episodic memory, which would not directly interfere with the participant’s naiveté, do not necessarily correlate with each other (Cheke and Clayton, 2013). Neither did the verbal IQ scores allow any conclusions on subsequent verbal episodic memory performance in our sample (ρ = -0.188, *p* = 0.233). Consequently, we assessed the baseline performance in the first immediate retrieval during the encoding phase as the moderator. The magnitude of activity in left prefrontal and temporal regions during the encoding phase has been shown to allow a prediction of subsequently remembered items (Rugg, 1998; Wagner et al., 1998; Iidaka et al., 2000). However, the number of words remembered during the first immediate retrieval of a verbal sequence that exceeds the participant’s working memory span does not necessarily equal the participant’s episodic memory ability as tested in the delayed recall. According to the serial position effect (Deese and Kaufman, 1957; Murdock and Bennet, 1962), the probability of recall for words at the beginning and the end of a presented list is elevated as opposed to items appearing in the middle of the list. Furthermore, different list segments have been linked to distinct cognitive abilities (Krueger and Salthouse, 2011), insofar as the primacy effect is connected with processing speed whereas the recency effect is explained by the affected material remaining in the short-term buffer and thus not requiring a separate retrieval operation to access it (Öztekin et al., 2010; Krueger and Salthouse, 2011). By contrast, the number of remembered midlist items allows drawing more consistent conclusions regarding an individual’s episodic memory ability (Krueger and Salthouse, 2011) and was thus the means of choice for assessing baseline performance in our study. Nevertheless, a confirmative moderation analysis demonstrated that the results did not differ substantially when all list segments were implemented.

Although ceiling effects were avoided during free recall, the latter became markedly apparent during the recognition task, irrespective of the stimulation group. On principle, it proved difficult to construct verbal learning tasks that sustain similar degrees of difficulty for both free recall and recognition as participants adjust their encoding procedures to subsequent demands (Hall et al., 1976) with free recall being experienced as more demanding as previous information needs to be recovered without prompts. As our task was primarily adjusted to the demands of the free recall condition, the recognition task was not sufficiently challenging for the healthy young participants. Consequently, the potential for improvement, a prerequisite for observable tDCS-derived benefits, was lacking and stimulation effects could not be verified.

Furthermore, acknowledging a tDCS-induced effect on basic cognitive processes as a distinct impact on high-level cognitive operations poses a potential problem. Notably, a previous fMRI study (Cabeza et al., 2003) exposed the common misconception that the observed activation pattern distinctly relates to the investigated task while neglecting to observe that the engagement in different tasks activates congruent brain regions. These considerations are particularly important with regard to prefrontal regions which are involved in controlling attentional processes (Rossi et al., 2009), also taking into account that episodic memory tasks require attention and thus may be only indirectly influenced. As alertness measures did not significantly differ between the real and sham group, nor correlated with performance during either encoding or retrieval phase, we could minimize the possibility that the observed tDCS effects on the number of remembered words were a mere consequence of a favorable stimulation impact on low-level attention. Given the dual attentional hypothesis (Cabeza, 2008), this may have been a confounding factor in studies that opted for the stimulation of the posterior parietal cortex (PPC) during the encoding phase of verbal learning paradigms (Jones et al., 2014; Manuel and Schnider, 2016) as the PPC is primarily involved in attentional processes that subserve memory rather than mnemonic functions *per se* (Berryhill, 2012). In spite of the absence of a relationship between attention and task performance in our study, the stimulation effects on other executive functions (e.g., working memory) that influence the performance in a verbal episodic memory task should be investigated in more detail in the future.

Owing to the experimental design in which we applied anodal tDCS for the entire duration of the encoding phase, the chosen moderator variable (i.e., midlist performance in the first immediate retrieval), which we used as a baseline measure, was already affected by the stimulation even though no significant group differences emerged in retrieval 1 [*t*_(40)_ = -1.677, *p* = 0.101]. Albeit stimulation effects appear only after approximately 5 min following the stimulation onset (Nitsche and Paulus, 2000), this timespan was already exhausted after the preceding alertness task. Therefore, future studies would require another approach to capture the baseline performance without interfering with the learning curve in the actual verbal episodic memory task.

Moreover, even though large inter-individual performance differences in the verbal episodic memory task were revealed, the collectively solid task performance of the study population might have prevented the manifestation of larger effects. Following those aging decreases cognitive abilities and that low performers in a given task are more likely to benefit from tDCS, larger effect sizes can be expected in older as well as in cognitively impaired populations, a notion that is backed by previous reviews (Tremblay et al., 2014; Hsu et al., 2015). While tDCS helped elderly participants to regain a brain activation pattern found in younger individuals (Meinzer et al., 2013), tDCS-induced benefits in healthy young adults seem to be limited by an inherent threshold, which requires further characterisation. Behavioral measures, however, might be too superficial to be used as a predictor whereas neurophysiological parameters like neurotransmitter levels and neural oscillations might represent better options (Habich et al., 2016). This is all the more relevant as previous studies in the primary motor cortex revealed positive correlations between the magnitude of the anodal tDCS-induced γ-aminobutyric acid (GABA) decrease and the performance in a force-adaptation task (Kim et al., 2014) and motor sequence learning, respectively (Stagg et al., 2011). As the main inhibitory neurotransmitter GABA is substantially involved in maintaining the balance of cortical excitation and inhibition (Isaacson and Scanziani, 2011). Consequently, a reduction of the GABA level leads to an increased excitability in the targeted task-related brain regions, supporting the intrinsic activity during task execution. On the other hand, increased excitability can also be detrimental when it exceeds the optimal range as it introduces additional noise to the system (Ozeki et al., 2009). However, how baseline performance measures, especially in cognitive domains, and subsequent tDCS benefits relate to initial GABA levels remains to be shown. Likewise, anodal tDCS has been indicated to enhance oscillatory activity in gamma- (Hoy et al., 2015), beta- (Mangia et al., 2014), alpha- (Spitoni et al., 2013), and theta-bands (Miller et al., 2015). Taking into consideration that cognitive deterioration in mild cognitive impairment has been associated with abnormal brain rhythms (Jelic et al., 2000; Nguyen et al., 2017), the favorable impact of tDCS on behavioral scores may also ensue from stimulation-induced changes in neural oscillation spectra. Nonetheless, the threefold connections between tDCS, brain rhythms and cognitive performance remain to be studied more systematically. Ultimately, these insights may not only help to select individuals according to fixed factors but also with regard to the introduction of customized closed-loop protocols to NIBS paradigms (Silvanto et al., 2008; Karabanov et al., 2016).

While this study centered on the improvement of verbal episodic memory via anodal tDCS, whose excitatory impact is clinically relevant, the comparison of behavioral effects of cathodal tDCS within the same paradigm might help to further explore the seemingly inherent restriction of beneficial stimulation outcomes to subgroups of the population.

While the present study did not include follow-ups on word list retrievals, the persistence of tDCS benefits is a pivotal criterion for its future application in a clinical context. So far, Sandrini et al. (2016) showed that benefits in verbal recall from a single session of anodal tDCS persisted until 48 h after stimulation while no stimulation-induced facilitation was found after 1 month. However, longer-term positive effects on naming performance in aphasic patients (Vestito et al., 2014) and performance in a visuospatial n-back task in healthy adults (Katz et al., 2017) were obtained with repeated sessions of anodal tDCS, with significant stimulation gains lasting up to several months. Even though only longer lasting effects would justify the application of tDCS as an efficient therapy option for cognitively impairment, future studies should also probe the duration of treatment effects after a single application of tDCS more extensively. These insights may provide information with respect to optimal spacing intervals between sessions, accomplishing cumulative stimulation benefits and simultaneously saving resources.

In summary, this study demonstrates that the proficiency in a specific task interacts with effects of tDCS on the performance in this task, with initially low performers profiting the most from real anodal stimulation. This insight implies that tDCS can improve memory functions only so far, suggesting a restriction of the treatment to cognitive underperformers.

## Author Contributions

JP, SK, ES, and CN designed research. AH performed research. AH, AA, and JP analyzed data. AH and JP drafted paper. SK, CN, ES, and AA revised paper critically.

## Conflict of Interest Statement

CN has received speaker honoraria from Vanda Pharmaceuticals. The other authors declare that the research was conducted in the absence of any commercial or financial relationships that could be construed as a potential conflict of interest.
